# ‘The current mental health status of children and young people with JIA, and their wider family’: a charity partner collaboration survey

**DOI:** 10.1186/s12969-023-00898-5

**Published:** 2023-10-06

**Authors:** Polly Livermore, S. Ainsworth, R. Beesley, S. Douglas, E. Earle, D. Wilson, L. Woolley, J. Clinch

**Affiliations:** 1 NIHR Advanced Clinical Academic Nursing Fellow, Infection, Immunity and Inflammation, Institute of Child Health, University College London Great Ormond Street Institute of Child Health, 6th Floor, 30 Guilford Street, WC1N 1EH London, UK; 2 Lupus UK, Essex, UK; 3 Juvenile Arthritis Research (JAR), Tonbridge, UK; 4 Scottish Network for Arthritis in Children (SNAC), Edinburgh, Scotland; 5 Children’s Chronic Arthritis Association (CCAA), Nuneaton, UK; 6 National Rheumatoid Arthritis Society (NRAS), Berkshire, UK; 7 Young People and Families, Versus Arthritis (VA), London, UK; 8 Bristol Royal Hospital for Children, Bristol, UK

**Keywords:** Paediatric rheumatology, Adolescent, JIA, Mental health, Support, Parents, Crisis, Psychology

## Abstract

**Background:**

This paper presents insight into the scale of mental health concerns for families who have a child or young person with a diagnosis of Juvenile Idiopathic Arthritis (JIA) living in any of the four nations of the United Kingdom (UK). The study’s objective is to share the current experiences of those that responded to a charity survey and consider future work to improve mental health support.

**Methods:**

This work was initiated and led by five UK charity partner organisations working with families affected by JIA. Parents/carers of a child or young person with JIA, and young people with JIA, submitted self-completion online questionnaires. The questionnaire asked 19 core questions, with a focus on the mental health impact of having and living with a JIA diagnosis. Questionnaires were delivered via charity partner UK-wide mailing lists and social media.

**Results:**

Questionnaire were completed by 291 participants over a 3-week period in February 2022. The majority of respondents were parents (229, 79%), 103 children had been diagnosed for over six years (35%), and 131 (45%) received shared care between paediatric rheumatology centres. In total, 168 (59%) children and young people with JIA had received, were currently receiving or were waiting for mental health support. Parents reported that their child’s diagnosis impacted their own mental health (218, 82%). Children and young people reported never being offered mental health support during appointments for JIA (157, 54%), and 71 (50%) of these had never received support.

**Conclusion:**

Children and young people with JIA have significant mental health sequelae from their diagnosis. Our findings found that nearly 60% of our respondents have had or are requiring mental health support, with significant numbers of parents/carers reporting difficulties in accessing care for their child’s mental health or their own mental health, due to their child’s diagnosis. This unique collaborative charity-led study, illustrates the importance of timely and accessible mental health support. Further work is needed to understand why best practice guidance for mental health support is not being met consistently and to identify how to embed it into standard rheumatology care.

**Supplementary Information:**

The online version contains supplementary material available at 10.1186/s12969-023-00898-5.

## Background

Mental health is defined as an individual’s cognitive, behavioural and emotional wellbeing, with mental health problems existing on a wide spectrum from low-grade anxiety to suicidal ideation [1]. In 2022, 18% of children aged 7 to 16 years and 22% of young people aged 17 to 24 years had a probable mental health disorder in England. These numbers have risen steeply in the last few years; for example, in 2017, only 10% of those aged 17–19 had a probable mental health disorder, compared to 18% in 2020 [2]. According to the Mental Health Foundation, 75% of these children and young people are not receiving the help they need to support good mental health [3]. Current waiting times are long and with many referrals to specialist services being inappropriately rejected, children and young people with diagnosable mental health conditions, including anxiety and depression, cannot access care [4].

At age 15, children with chronic health conditions are 60% more likely to be diagnosed with mental health disorders compared to than healthy peers [5]. Mental health problems, particularly depression and anxiety, have been shown to be more common in children with rheumatological disease than in their healthy peers [6, 7]. Children and young people with paediatric rheumatological conditions may suffer from significant psychological burden associated with adverse outcomes, including decreased quality of life, increased pain and disease activity, physical disabilities, school absenteeism due to disease flares and treatments, suboptimal medication adherence and challenges associated with transition to adult care [8].

The impact of chronic disease on mental health is known to affect the wider family. Caregivers of children and young people with JIA identified the following wider concerns: their own mental health, coping skills, economic impact, family roles, impact of diagnosis, their own relationships, impact at work and knowledge of the disease [9]. Children’s anxiety and depressive symptoms moderately correlate with those of their parents, with 12% of parents having clinically significant depressive symptoms [10]. Uncertainty can also impact parental coping, with parents reporting uncertainty that could not be resolved with information alone [11].

The World Health Organisation guidelines for chronic pain in childhood 2021, a symptom that affects many children and young people with rheumatological conditions, recommend three areas of interventions: physical therapy, pharmacological management and psychological therapy [12]. Furthermore, the 2010 British Society of Paediatric and Adolescent Rheumatology (BSPAR) Standards of Care [13] (now merged into the British Society of Rheumatology (BSR)), also recommend that a paediatric clinical psychologist should be part of every paediatric rheumatology multidisciplinary team. Despite this, few paediatric rheumatology units have adequate or any resource to provide such support, with 47% having no named psychologist as part of their rheumatology team [14]. The 2019 BSR State of Play report [15] showed poor access to psychology expertise and recommended ‘increased provision of all members of the multidisciplinary team with an emphasis on increased psychology provision and group psychology sessions’. However, in practice, with such poor support, children and young people with rheumatology conditions who do not have access to mental health services at their paediatric units are generally being signposted to local Child and Adolescent Mental Health Services (CAMHS). At present, whilst waiting lists vary across the country, the median waiting time for treatment across mental health providers varies from 1 to 182 days (6 months) [16]. In addition, there were 24% more patients in contact with CAMHS in 2021 than in 2020 and 44% more than in 2019. These figures include those waiting to be seen, suggesting that CAMHS may be struggling to meet the growing demand [17].

Anecdotal reports highlighted concerns about mental health provision for children and young people with JIA, exacerbated by National ‘Standards of Care’ Guidelines [12] not being universally implemented. Initially, a fact-finding conversation by a number of paediatric rheumatology charities, driven by concerns raised by families, sought to identify potential ideas to improve the current landscape of care. At the time, the COVID-19 pandemic compounded increasing mental health difficulties nationally, especially for children and young people. This led to a decision to work together as a group of charities to collect children and families experiences and share the current situation of mental health support for paediatric rheumatology in the UK. A collaboration of five UK partner charities (Children’s Chronic Arthritis Association (CCAA), Juvenile Arthritis Research (JAR), National Rheumatoid Arthritis Society (NRAS), Scottish Network for Arthritis in Children (SNAC) and Versus Arthritis (VA)), representing children and young people with JIA, conducted this study to identify the extent of this issue and to better understand the need. A named individual represented each partner charity, all of which were engaged and supportive of this work.

This survey was supported by the British Society of Rheumatology (BSR), represented in meetings by a Consultant Paediatric Rheumatologist (JC) and a Paediatric Rheumatology Senior Nurse Matron (PL). The overall objective of this study was therefore to present the current mental health experience and needs of children, young people and their families living with JIA in the UK. This is the first paper to share such views led by a charity consortium, with the aim of raising awareness of the need to do more to support mental health in paediatric rheumatology.

## Methods

The survey was designed by the partner charities, who brainstormed key objectives and developed questions to address them. It was therefore not validated, but it was piloted with a small group of charity identified partners. The final survey consisted of 19 core questions, with some routing depending on answers. Demographic data were kept to a minimum (child/young person’s age, time since diagnosis, region of UK and structure of JIA care, e.g. shared care/secondary care only etc.), with no identifiable data requested (see Supplementary Data 1 for full survey). The majority of questions were closed-ended multiple choice, with some open text comment options. for example ‘Does your hospital team have access to mental health support services for you or your child/young person if you need them? Yes/No/Don’t know and (if answered yes to being on a waiting list), ‘How long in months have you been on the waiting list for? with free text response’. The survey was advertised through charity mailing lists and social media channels and was open from 7th to 25th February 2022. Included were three distinct response options: parents/carers of a child or young person between the ages of 0–18 with JIA; dyad completions with children with JIA and their parent or carer; and young people with JIA. We requested that there was only one completion per child/young person with JIA.

### Ethics

This was a charity-led study comprising an online, self-referred anonymous questionnaire, NHS ethical approval was therefore not required, as confirmed by NHS REC online tools (http://www.hra-decisiontools.org.uk/ethics). However, good ethical practice was followed at every stage. Each family was asked to give consent to take part at the time of completion, and could not enter the questionnaire without doing so. All participants were asked to not add any identifiable information in the open text comments. Due to the personalised and highly sensitive nature of this survey, all quotes reported here are completely anonymous, kept to a minimum, any possible identifiable information has been deleted or altered and participant responses are not numbered, to ensure that all data are completely unidentifiable.

### Analysis

Quantitative data were analysed descriptively, showing total numbers and percentages firstly for the demographic data collected, followed by the answers to closed questions. Reflexive thematic analysis was used for open text comments using an inductive and data-driven approach to identify semantic themes. Braun and Clarke’s, six analytical phases were followed; (1) dataset familiarisation, (2) data coding, (3) initial theme generation, (4) theme development and review, (5) theme refining, defining and naming and (6) writing up [18, 19]. Initial analysis was undertaken by the rheumatology matron (PL), who is an experienced qualitative researcher. All themes were discussed with the steering group and validated to develop a set of consistent codes and themes that were used to represent the final data. Data presentation displays in turn the demographic data, quantitative data and qualitative data.

## Results

In total, there were 291 respondents, with no repeats when the core demographic data was combined, (such as location, age of child, structure of healthcare and disease duration), implying that there were no repeated entries from the same individuals in one household. Demographic data are shown in Table 1. Respondents were from all geographical regions across the UK, with the largest proportion living in Scotland (60, 21%) followed by the South-East of England (45, 15%). Time spent on a waiting list for the 29 (11%) who reported being on a list, ranged from 1 to 24 months, with a range of comments, with 5 reporting 12 months, some just saying “months” and some saying “unsure”. The largest majority of patients (35%) had been diagnosed for longer than 6 years.

**Table 1:** Demographic and clinical care details

			Number
**Questionnaire completed by**			
Parent	229 (79%)		
Child or Young person (12–18 years)	44 (15%)	12-14yrs	6
		15yrs	9
		16yrs	8
		17yrs	10
		18yrs	11
Parent & child dyad	18 (6%)		
Total	291 (100%)		
**Age of child or young person**		0-18yrs (median 12)	(8.5, 15) IQR
**How long is it since you or your child/young person was diagnosed?**			
Within the last year			42 (14%)
1–3 yrs ago			81 (28%)
4–6 yrs ago			65 (22%)
Longer than 6yrs ago			103 (35%)
Total			291 (100%)
**Which structure of care best describes the type of care you or your child / young person receives for your/their JIA?**			
Shared care between local & specialist hospital			131 (45%)
Care from local paediatrician only			19 (7%)
Rheumatologist at an adult clinic			28 (10%)
Specialist hospital – no local care			73 (25%)
Other			38 (13%)
Total answered			289/291 (99%)

### Quantitative data

Table 2 shows responses to all the closed questions. Respondents were asked to select an option regarding their child’s, or their own (if a young person), current situation regarding mental health support. Of the 283 responses, 115 (41%) stated that their child/young person have not needed mental health support. Of the remaining 168 (59%), 62 (22%) children and young people have previously had, 51 (18%) are currently having or 55 (20%) are waiting for mental health support. There were free text comments from families who shared that they need intervention, but had not yet received any and were also not on a waiting list (as evidenced by some of the comments received). There may be some in this category who have not yet asked for help, but recognise that intervention is needed. Of the 8 non-respondents, 6 were parents and 2 were a parent and child dyad completion; all of these reported that they had never been offered access to mental health support services, they were all unaware whether their hospital team had access to mental health services, and all responded that their child’s diagnosis, had impacted their own mental health.

**Table 2:** Responses to closed-ended questions

**Does your hospital team have access to mental health support services for you or your child/young person if you need them?**	
Yes	116 (40%)
No	22 (8%)
Unaware	153 (53%)
**Total**	**291 (100%)**
**Have you or your child/young person been offered access to mental health support services during appointments for JIA?**	
Yes	106 (36%)
No	157 (54%)
Not sure	28 (10%)
**Total**	**291 (100%)**
**If no, did you or you child/young person get mental health support from somewhere else?**	
Yes	28 (20%)
No	71 (50%)
Have not needed mental health support	42 (30%)
**Total**	**141**
**If you are a parent/carer do you think your family’s mental health has been impacted by your child or young person’s diagnosis? (respondents could select for both parents and siblings Y/N and not sure)**	
Yes impact on parents	218 (82%)
Yes, impact on siblings	126 (47%)
No impact on parents	18 (7%)
No impact on siblings	19 (7%)
Not sure	26 (10%)
**If yes to PARENTS, have you been able to access relevant and suitable mental health support?**	
Yes	31 (14%)
No	59 (27%)
I have not tried to access support	100 (46%)
Did not answer	28 (13%)
Comments	27
**If yes to SIBLINGS, have you been able to access relevant and suitable mental health support for them?**	
Yes	14 (11%)
No	32 (25%)
I have not tried to access support	68 (54%)
Did not answer	12 (10%)
Comments	10
**What is the current situation of you or your child/young person regarding mental health support?**	
Waiting to go on a waiting list	25 (9%)
On a waiting list	30 (10%)
Being seen	51 (18%)
Finished care sessions	62 (21%)
Have not needed mental health support	115 (40%)
Did not answer	8 (3%)
Total who answered	283

Overall, over half (153, 53%) of respondents reported that they were unaware whether their hospital team had access to mental health support services if needed, with 116 (40%) saying yes. When asked if they had been offered mental health support during appointments, 157 (54%) replied ‘no’. A further question specifically asked these respondents, if they had received mental health support from somewhere else, with 71 (50%) again replying no. There were 20% who had been offered support, with 105 free text answers: 52 (51%) mentioned a psychologist and 15 (14%) mentioned ‘Other’, with a range of answers including still waiting for support, and gradual exposure therapy. The other 30% said they had not needed mental health support. When asked where they had received help from if not during an appointment, there were 54 free text comments, with the highest proportion (9, 17%) citing CAMHS, 7 (13%) saying school or college, and fewer respondents mentioning GP, parent or local support groups.

When asked if their family’s mental health had been impacted by the diagnosis of JIA, 218 (82%) parents said it had impacted them. Of these, 100 (46%) had not tried to access support, 31 (14% however replied yes, and 59 (27%) replied no, they had not been able to access support. Parents reported in 126 (47%) cases that they felt that the JIA diagnosis had impacted their siblings’ mental health, with 100 (46%) of these not trying to access support for help.

**Fig. 1:**
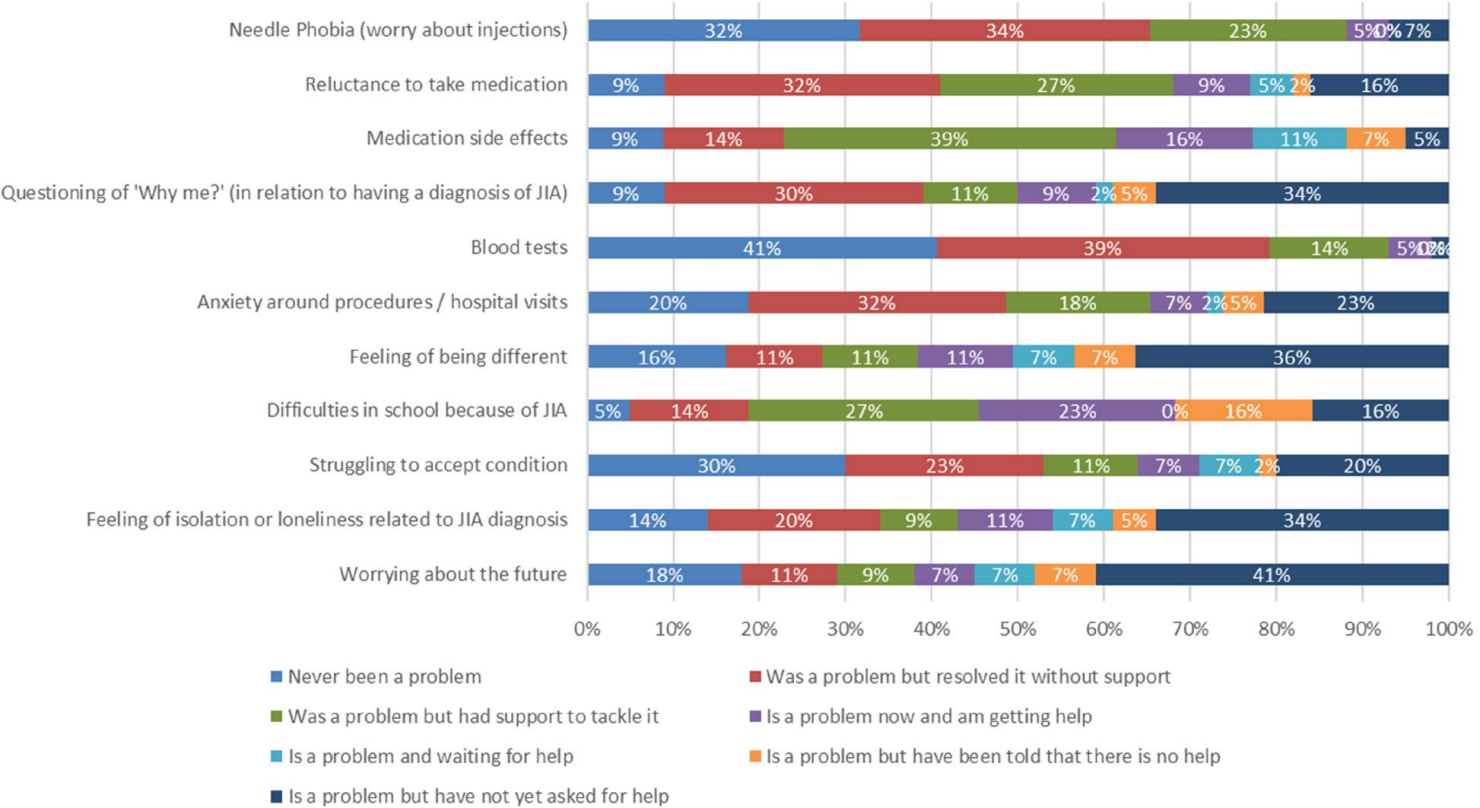
Stacked barchart to illustrate JIA disease specific influences on mental health of young people’s (*n* = 44) responses only

Every respondent was asked specifically about issues related to their (or their child or young person’s) JIA diagnosis and whether these had been or are currently a problem (Fig. 1). Overall, 235 (81%) respondents felt that ‘worry about injections’ had been an issue at some point, and 233 (80%) had been or are ‘reluctant to take medications’. Emotions such as ‘Questioning why me’ and ‘Feelings of being different’ also occurred in a significant number of responses, affecting 219 (75%) and 213 (73%) respectively.

**Fig. 2:**
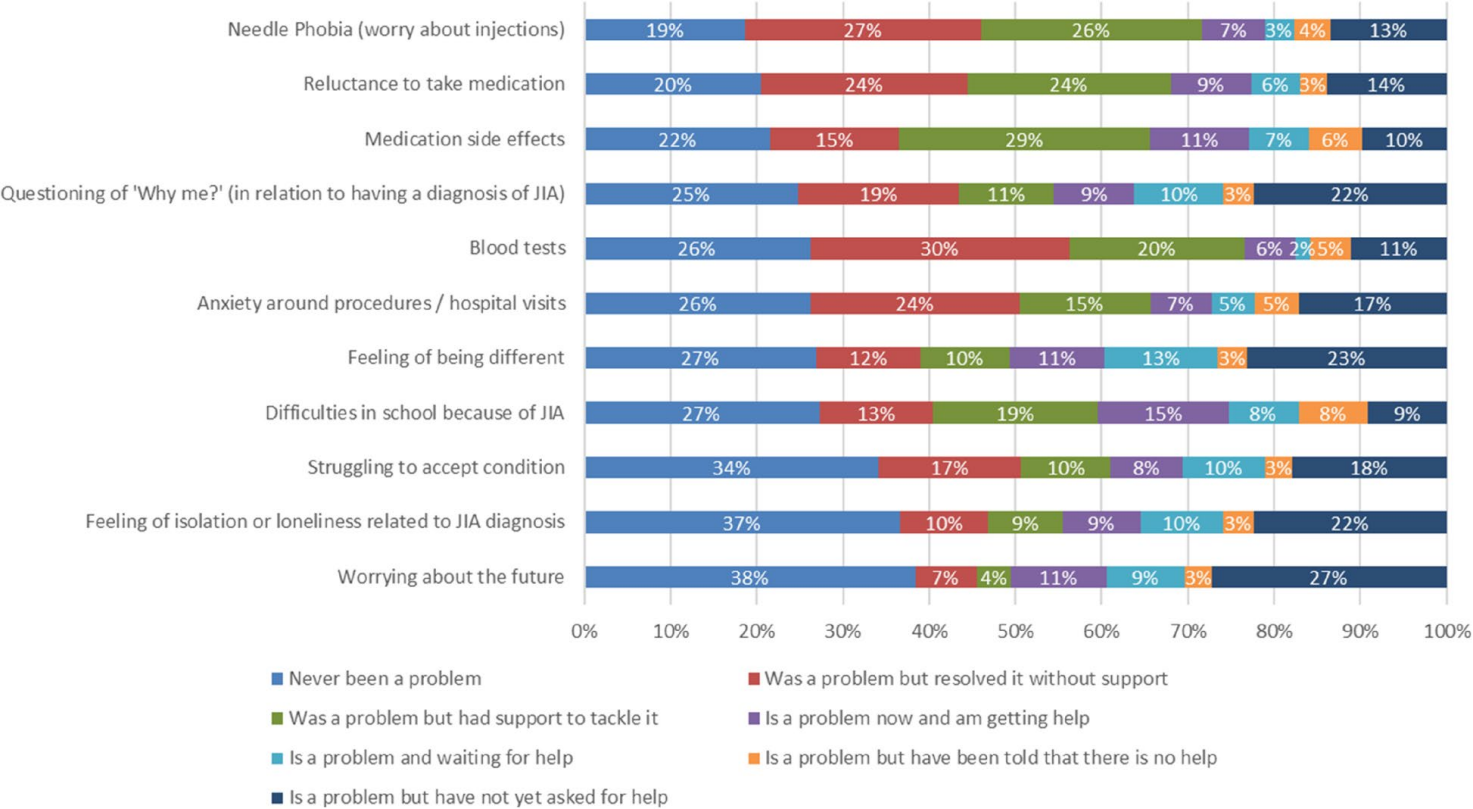
Stacked barchart to illustrate JIA disease specific influences on mental health, all 291 respondents (Parents/children/young people)

When the 44 children and young people’s independent self-report data are plotted (Fig. 1), there is a clear discrepancy compared to that of all respondents combined (Fig. 2). For example, only 5% have never had any difficulties in school because of their JIA compared to a result of 27% for parent and parent and child/young person completion together. Additionally, 41% of young people are currently worrying about the future, but have not yet asked for help with this, whereas in the combined result this was much lower at 27% of respondents.

### Qualitative data

There were two questions where families could input open text responses, the first asked - ‘any other issues related to managing JIA that they would like to share’; the other asked - ‘whether there was anything else they would like to share about their experience of mental health support within paediatric rheumatology’. For these two questions, there were 187 free text comments, with only 14 from young person self reports. Six themes were identified, each of which will be presented here in turn:

#### Impact from having JIA

Impact from having JIA was the largest theme, and encompassed three sub-themes: rheumatology specific impact, current mental health and individual needs. Rheumatology specific issues mentioned included: the impact of methotrexate, needle aversion, fatigue, inability to do sports, and pain. Parents particularly talked about the impact of regular injections: “he is only 5 and becoming very angry about needles and tests” and “their anxiety about injections is getting worse”. Parents openly shared their own feelings of helplessness: “at a loss of how best to help and support my child” and “we need help. I have asked, but no real help offered”. Young people who self completed the survey were more inclined to detail the effects of their JIA on their mental health, such as “feeling of loneliness one feels with JIA” and “low mood”. One parent talked about current mental health by highlighting parity of esteem by saying, “our child’s mental health suffered from the onset of symptoms to present day. Ongoing anxiety around school, friendships, loneliness. I believe mental health support should be just as important as physical health”. Some parents highlighted specific individual needs, such as the impact of JIA in conjunction with a concurrent condition, for example “having Autism as well as JIA has been difficult. The struggles of having JIA are amplified by not being able to understand and deal with how you’re feeling”.

#### Impact upon the family

Not surprisingly, due to the family focus of these questions, parents/carers shared how they are functioning as a family and the effect their child’s diagnosis has had on their own lives. The impact upon parents’ own mental health was detailed within some of the quotes, “parents need support to support their child”, “it caused a marriage breakdown”, “support for parents dealing with how the child is feeling and their reluctance to accept medication, condition etc” and “I feel like as my child is so young there is so many questions, I feel upset about the whole thing but I have to carry on and be strong for my child even when I just ask why this has happened to my baby”.

These two open ended questions gave parents an opportunity to talk about the impact on their siblings. Four parents choose here to add free text comments, for example, “sibling has severe jealousy” and “daughters needle phobia created a needle phobia for her sister!”.

#### Social impact

There were three sub themes within social impact: Impact upon education, Impact upon employment and Peer relationships. Within the sub theme of education, the role parents adopt as their child’s advocate was emphasised, with parents feeling that they are responsible for imparting information and speaking up when they feel their child is disadvantaged or disbelieved: “Making school aware that mental health worries can happen along with physical illness because our school don’t believe there is any anxiety”and “they moan about her attendance even though they know it is due to hospital appointments. She misses out on attendance-related prizes”.

There were fewer employment comments highlighted, but the ones that were mentioned were done so by parents/carers and highlight the uncertain future, such as “I worry about job capability”. Peer relationships included concerns about: “the impact of peers not understanding and feeling separated from them”, “body image and self harm, relationship problems for teenagers” and “managing JIA and social life, managing JIA while in transition years in school and going to university”.

#### Crisis mental health

While there were few (11) mentions of children and young people who had tried or openly communicated that they wanted to take their own life, from 11 separate families, these unprompted comments were very powerful and clearly illustrate an urgent need to further support these families. Parents shared “would help if offered mental health support when medication started. Only getting help as took paracetamol” and “suicide attempt 12 months after starting rheumatology medication, currently under CAMHS”.

#### Gaps in care

Gaps in care was the biggest theme in relation to the question asking “Is there anything else you would like to tell us about your experience of mental health support within rheumatology?” with 45 out of 88 free text responses being attributed to this theme.

There were various mentions of gaps in care resulting in six sub themes: Lack of holistic care, Timely care, Impact due to delay, Lack of understanding from health care professionals, Impact upon transition and Lack of support. Lack of holistic care included concerns such as: “my son has a number of autoimmune conditions and there is a lack of joined up thinking around this”, “difficulties with shared care when both hospitals are hours away from each other” and “services are spread”. Timely care included: “Instant access at time of issues is essential”, “the importance of care when it is needed”, and “I think parents / children should be provided with help via hospital rather than having to wait on a huge waiting list for CAMHS”. Impact due to delay highlighted that: “excellent rheumatology team but think mental health provision should be offered as part of treatment. We had to go elsewhere. This added to the problem by delaying treatment” and “waiting for a diagnosis caused problems”.

An example of lack of understanding from health care professionals includes: “Professionals not believing how much pain the child is in and not believing the symptoms that the parents/child say they have. This leads the child to feel like what is the point in telling anyone because nobody will believe me!”. Impact upon transition was an important point specifically for the young people who completed the questionnaires themselves, which included comments about: “A mid teen diagnosis meant I’m on the cusp of adulthood so got caught between services and never got the school related support you’d get from a children’s hospital”, “remember that teenagers need to be treated differently to younger children in the support they need and issues they face” and “going straight into adult care with no support as a mid-teenager”. Lack of support was the last sub-theme: “never been offered support, told it is normal”, “had help, but didn’t help” and “I’m not clear what is available and what the thresholds for accessing the waiting list/treatment are”.

#### Good experiences

Some families choose to share their examples of good care, in particular, the support received from paediatric rheumatology charities and other providers: “family days have been invaluable” and “peer support from other parents have really helped”. Examples were also provided of charities who had been particularly invaluable while children and young people waited for help, either from their clinical teams or in the community.

## Discussion

This study provides the first analysis of family-reported experiences of mental health challenges associated with a diagnosis of JIA in the UK. Whilst these results came from families living in the UK who responded to a charity led-survey, the two findings of particular importance: (i) the impact on parental mental health and (ii) the need for mental health support for the child or young person themselves, are relevant to any family living with JIA, in any culture or any country.

In this survey, the majority of parents said their child’s diagnosis had impacted their own mental health. The closest report in the literature looking at parental mental health is a study by Vuorimaa et al. (2011), who found that 12% of their parents had clinically significant depressive symptoms and 7% had anxiety [10]. While we did not specifically ask about depression and anxiety, these figures are significantly less than the 82% of parents in our study who responded yes when asked if their own mental health had been impacted by their child’s JIA diagnosis. Russo (2012) found that 17% of the mothers of children with JIA in their study had sought psychological support and a quarter reported being on anti-depressants [20]. Our qualitative findings mirror this report, with some parents openly sharing their own challenges and sharing the help they need. Parents were similarly asked whether they thought the JIA diagnosis had impacted siblings’ mental health, with 47% saying yes. While there was no ability to record that there were no siblings, with only 7% saying no for parents and 7% saying no for siblings, these results are quite startling. The only other study identified in this area reported that 33% of siblings of 33 children and young people with JIA had sought psychological support; however, the overall number of siblings was not reported, thus making interpretation of this figure difficult [20].

Fair et al. (2022) recently demonstrated that approximately one quarter of children with JIA in their American cohort reported moderate to severe symptoms of anxiety and depression [7]. This supports our finding that a significant proportion of children and young people with JIA are suffering with their mental health and that we need to do more world-wide to address these figures. This is the first time we have been able to clearly describe the magnitude of children and young people in the UK with JIA, who need further psychological input. These findings can help raise awareness and champion further psychological support for those with JIA.

The survey asked about particular aspects related to having a health condition, to see the impact these had on children and young people, including; needle phobia, reluctance to take medicines, blood tests and anxiety around procedures. Not surprisingly, the impact of having JIA was the biggest theme identified through the qualitative analysis, with some comments raised in relation to JIA specifically. These included: the psychological impact from not being able to keep up with sport and hobbies, fatigue, pain, loneliness and the negative impacts of their weekly methotrexate injection, including in some – phobia of the colour yellow. These issues are important when considering where is the best place to manage mental health concerns – within local mental health services or within rheumatology services. Local psychology services may not understand the rationale behind some of these concerns, and thus, they may be best treated within paediatric, or adolescent rheumatology centres. It is also worth considering how many of the issues raised within the survey responses are predictable and common for children and young people with rheumatic conditions. Through offering pre-emptive, preventative care, many of these issues might be prevented from escalating, developing and potentially spiralling into severe mental health issues by the provision of timely support from qualified professionals.

Although the number of young person self-completion respondents was small (44, 15%) the differences in responses to parent completion are important. There were much fewer free text responses, even though the scoring showed greater challenges with rheumatology-related questions (Fig. 1). This discrepancy is well described in the literature and highlights the importance of ensuring that young people have an opportunity to speak on matters relating to their own health [21].

Gaps in care was a frequently reoccurring theme in the qualitative responses. Families felt that if health care professionals had offered more timely support when it was needed, specifically for rheumatology-specific issues, then the burden families highlighted may have had less impact upon them.

While the total number of respondents referring to crisis mental health only made up a small proportion of respondents, these were unprompted and so are likely to underestimate the scale of the issue. Signposting to specialist services and back to health care providers is outside paediatric rheumatology charity remits; however, daily conversations with families highlight unmet need and delays in access to appropriate care. Ensuring that timely support is available for families who need it, when they need it, should be embedded into all paediatric rheumatology care worldwide.

### Limitations

As this survey was driven by a collaboration of charities working with families and young people living with JIA, there is an inherent bias from the families who would have had access to the questionnaires. Those linked in with UK charity networks may have different characteristics from those who are not, for example, those families who had already actively sought out support. There were a number of responses throughout the survey that praised the charities for their support of families with a JIA diagnosis, so it may be, that the responses here, present a more positive picture of mental health support in families than is actually the case. With any survey, bias is also unavoidable from those who complete the survey as it personally resonates with them, often from experiencing challenges themselves or receiving care in this area. Therefore the results here may be unrepresentative of the population as a whole. Similarly, it is difficult to make comparisons to the published literature already available as our study did not use standardised questionnaires or have healthy controls. As highlighted earlier, unfortunately, not all questions captured all possible answers (for example the sibling question, only asked if siblings had been affected yes or no, not offering an option for if there were no siblings), and the survey was only accessible to those who could understand written English. It was also anticipated that the younger age range of children, possibly preschool age, may be likely to have fewer challenges to their mental health due to their developmental level and time since diagnosis. This was supported by some of the free text comments that mentioned both being newly-diagnosed or being a young age; some of these issues had not yet become a concern. However, as one parent commented, by thinking about some of these issues now, they may be able to prevent them from occurring in the future. Young people over 18 years of age with JIA were not included in this study, nor were those with other rheumatic conditions. There are plans to offer the survey to these groups in the future.

## Conclusion

This is an interesting study for paediatric rheumatology with the formation of a multi-partner charity collaboration to join forces to raise the issue of mental health in Juvenile Idiopathic Arthritis. Whilst we have detailed the mental health challenges that so many families of children and young people with Juvenile Idiopathic Arthritis across the UK are experiencing today, these results may be applicable to any child or young person with JIA in any country. We have also demonstrated through a combination of statistics and powerful comments, how too many families are suffering without adequate timely, tailored mental health care. This work has provided preliminary evidence to take forward a campaign to firmly put mental health in paediatric rheumatology in the spotlight. Work has begun by involving other paediatric rheumatology charities in the UK and beyond, to embrace all paediatric rheumatology conditions, and a planned webinar to highlight specific areas of support and collaboration with mental health charities. Understanding the bigger picture of the frustrations, challenges and good practice facilitators is vital to work out how to move forward.

This study has served to illustrate that promoting good mental health, should always be part of general rheumatology medical care [6] on par with physical treatment. Timely and accessible access throughout the life course, pre-emptive in all cases, especially when it is needed, is vital. This is critical to achieving improved and equitable outcomes for all children, young people and their families, and to ensure that country specific care standards are achieved.

### Supplementary Information

**Additional file 1: Supplementary Data 1.** Mental Health Survey questions

## Data Availability

The datasets (surveys) analysed during the current study are not publicly available as there is the risk of patient identification but are available from the corresponding author on reasonable request.

## Abbreviations

BSPAR: British Society of Paediatric and Adolescent Rheumatology
BSR: British Society of Rheumatology
CAMHS: Child and Adolescent Mental Health Services
CCAA: Children’s Chronic Arthritis Association
JAR: Juvenile Arthritis Research
JIA: Juvenile Idiopathic Arthritis
NRAS: National Rheumatoid Arthritis Society
SNAC: Scottish Network for Arthritis in Children
UK: United Kingdom
VA: Versus Arthritis
