# Supplementary material for: ‘The current mental health status of children and young people with JIA, and their wider family’: a charity partner collaboration survey
Source: Pediatr Rheumatol Online J. 2023 Oct 6;21:111. doi: 10.1186/s12969-023-00898-5 (PMC10557198; doi:10.1186/s12969-023-00898-5)
Supplement: Supplementary file 1 — Additional file 1: Supplementary Data 1. Mental Health Survey questions [file 12969_2023_898_MOESM1_ESM.docx]

**Supplementary Data 1**

**Mental Health Survey questions**

1.NRAS are hosting the survey and the data will be shared with Polly Livermore a Professional NRAS Volunteer, member of BSR and Lead Paediatric and Adolescent Nurse Representative from the UK. However, please note we do not ask for any data that can be identifiable to you or your child. If you are happy for this, please click to give your consent.

2.How old are you or your child/young person who has JIA?

3.We are keen to understand who is completing this survey. Are you:Parent/Young person/Parent and young person

4.How long is it since you or your child/young person was diagnosed?

5.Which region of the United Kingdom do you live in?

6.There are different ways in which JIA care is structured in the UK. Which of these best describes the type of care you or your child/young person receives for your/their JIA?

7.Does your hospital team have access to mental health support services for you or your child/young person if you need them?

8.Have you or your child/young person been offered access to mental health support services during appointments for JIA?

9.If yes what type of support were you or your child/young person offered or who provided this support? Please do NOT provide any personal names or contact details

10.If no, did you or your child/young person get mental health support from somewhere else?

11.If yes please tell us where you or your child/young person received this mental health support Please do NOT provide in any personal names or contact details

12.What is the current situation of you or your child/young person regarding mental health support?

13.How long in months have you been on the waiting list for?

14(a). We know that certain issues related to managing JIA can be difficult for some children and young people. Please tell us about you or your child/young person’s experience of these particular issues. Please select the most appropriate option for each: Depending on your device you may have to scroll right and back again with the bar at the bottom of the question table: Needle Phobia (worry about injections)

14(b). We know that certain issues related to managing JIA can be difficult for some children and young people. Please tell us about you or your child/young person’s experience of these particular issues. Please select the most appropriate option for each: Depending on your device you may have to scroll right and back again with the bar at the bottom of the question table: Blood tests

14(c). We know that certain issues related to managing JIA can be difficult for some children and young people. Please tell us about you or your child/young person’s experience of these particular issues. Please select the most appropriate option for each: Depending on your device you may have to scroll right and back again with the bar at the bottom of the question table: Reluctance to take medication

14(d). We know that certain issues related to managing JIA can be difficult for some children and young people. Please tell us about you or your child/young person’s experience of these particular issues. Please select the most appropriate option for each: Depending on your device you may have to scroll right and back again with the bar at the bottom of the question table: Medication side effects

14(e). We know that certain issues related to managing JIA can be difficult for some children and young people. Please tell us about you or your child/young person’s experience of these particular issues. Please select the most appropriate option for each: Depending on your device you may have to scroll right and back again with the bar at the bottom of the question table: Feeling of being different

14(f). We know that certain issues related to managing JIA can be difficult for some children and young people. Please tell us about you or your child/young person’s experience of these particular issues. Please select the most appropriate option for each: Depending on your device you may have to scroll right and back again with the bar at the bottom of the question table: Questioning of 'Why me?' (in relation to having a diagnosis of JIA)

14(g). We know that certain issues related to managing JIA can be difficult for some children and young people. Please tell us about you or your child/young person’s experience of these particular issues. Please select the most appropriate option for each: Depending on your device you may have to scroll right and back again with the bar at the bottom of the question table: Struggling to accept condition

14(h). We know that certain issues related to managing JIA can be difficult for some children and young people. Please tell us about you or your child/young person’s experience of these particular issues. Please select the most appropriate option for each: Depending on your device you may have to scroll right and back again with the bar at the bottom of the question table: Feeling of isolation or loneliness related to JIA diagnosis

14(i). We know that certain issues related to managing JIA can be difficult for some children and young people. Please tell us about you or your child/young person’s experience of these particular issues. Please select the most appropriate option for each: Depending on your device you may have to scroll right and back again with the bar at the bottom of the question table: Anxiety around procedures / hospital visits

14(j). We know that certain issues related to managing JIA can be difficult for some children and young people. Please tell us about you or your child/young person’s experience of these particular issues. Please select the most appropriate option for each: Depending on your device you may have to scroll right and back again with the bar at the bottom of the question table: Difficulties in school because of JIA

14(k). We know that certain issues related to managing JIA can be difficult for some children and young people. Please tell us about you or your child/young person’s experience of these particular issues. Please select the most appropriate option for each: Depending on your device you may have to scroll right and back again with the bar at the bottom of the question table: Worrying about the future

15.Apart from the above are there any other issues related to managing JIA that you think we should be aware of? Please do NOT provide any personal names or contact details.

16.If you are a parent/carer do you feel your family’s mental health has been impacted by your child/young person’s JIA diagnosis?

17.If yes to parents, have you been able to access relevant and suitable mental health support? Please do NOT provide any personal names or contact details.

18.If yes for siblings, have the sibling(s) been able to access relevant and suitable mental health support? Please do NOT provide any personal names or contact details.

19.Is there anything else you would like to tell us about your experience of mental health support within rheumatology? Please do NOT provide any personal names or contact details.
